# Molecular Dynamics Simulation on the Conformational Transition of the Mad2 Protein from the Open to the Closed State

**DOI:** 10.3390/ijms15045553

**Published:** 2014-03-31

**Authors:** Chaoqun Li, Yanyan Zhu, Yan Wang, Guangju Chen

**Affiliations:** 1 College of Chemistry, Beijing Normal University, 19 Xinjiekouwai Street, Beijing 100875, China; E-Mail: lichaoqun_210@163.com; 2 Department of Chemistry, Zhengzhou University, Zhengzhou 450052, Henan, China; E-Mail: zhuyan@zzu.edu.cn

**Keywords:** molecular dynamics simulations, open/closed Mad2, dynamical transition mechanism

## Abstract

The Mad2 protein, with two distinct conformations of open- and closed-states, is a key player in the spindle checkpoint. The closed Mad2 state is more active than the open one. We carried out conventional and targeted molecular dynamics simulations for the two stable Mad2 states and their conformational transition to address the dynamical transition mechanism from the open to the closed state. The intermediate structure in the transition process shows exposure of the β6 strand and an increase of space around the binding sites of β6 strand due to the unfolding of the β7/8 sheet and movement of the β6/4/5 sheet close to the αC helix. Therefore, Mad2 binding to the Cdc20 protein in the spindle checkpoint is made possible. The interconversion between these two states might facilitate the functional activity of the Mad2 protein. Motion correlation analysis revealed the allosteric network between the β1 strand and β7/8 sheet via communication of the β5-αC loop and the β6/4/5 sheet in this transition process.

## 1. Introduction

The spindle assembly checkpoint (SAC) proteins monitor a series of events in mitosis to ensure the accuracy of chromosome segregation prior to cell division, and are indispensable for chromosome stability [1–4]. Mitotic arrest deficiency 2 (Mad2), which is a critical molecular component of the SAC proteins, is able to inhibit the anaphase-promoting complex or cyclosome (APC/C), combining with Cdc20 (an activator of APC/C) to delay separation of premature sister-chromatid through the binding of Mad2 to Cdc20 [5–14]. Premature sister-chromatid separation leads to aneuploidy (abnormal number of chromosomes), which contributes to cancer progression [5,15–18]. Mad2 binding to Cdc20 is helpful for accurate chromosome segregation to prevent cancer development. Therefore, it is important that the active Mad2 protein binds to Cdc20 in the mitotic process.

Usually, a given protein has a unique and native three-dimensional structure with the lowest possible Gibbs free energy [19]. However, Mad2 shows the characteristics of a “metamorphic” protein, and is able to adopt two distinctly different conformations, *i.e.*, the open and closed Mad2 states under native conditions [20–22]. The first high-resolution X-ray crystal structure of the open Mad2 state was determined by Luo *et al.* in 2000 [23]. Since then, significant progress has been made in filling the structural space of this family. Luo *et al.* again reported the structures of several closed Mad2 states in 2002 and 2004 [20,24]. Subsequently, other crystal structures of the Mad2 monomer and dimer were also reported [14,25,26]. These crystal structures revealed that the structure of the closed Mad2 state is strikingly different from the open Mad2 state. In the open Mad2 state, the structure of Mad2 consists of three layers: a central layer formed by three α-helices (αA, αB and αC), a large six β-sheet ordered as β7–β8–β6–β4–β5–β1 in the front layer, and a short β-hairpin (β2, β3) in the back layer. In the closed Mad2 state, the predominant differences in features of this state with respect to the open one are the large five β-sheet ordered as β6–β4–β5–β8″–β8′ and a *N*-terminal β1 strand forming an additional helix attached at the αA helix. Moreover, the β6 strand is exposed in the closed Mad2 state, while it is blocked in the open Mad2 state. It has been reported that Cdc20 easily forms an edge-on interaction with the β6 strand at the ligand-binding site in the closed Mad2 state. The closed Mad2 monomer or dimer is more functionally active than the open one, which might facilitate Cdc20 binding in order to impart fidelity chromosome segregation.

Cytosolic Mad2 in human cells is mainly represented by an open Mad2 monomer [20]. Then, the active closed Mad2 conformation should be initially transformed by the open Mad2 monomer with or without the Mad1–Mad2 template [27–31]. Therefore, the conversion of the open state to the closed state in the Mad2 protein plays a key role for Cdc20 binding and has become an area of considerable interest over the past decades. It is well-known that the open Mad2 state is able to spontaneously convert to the closed Mad2 state [20]. Based on the difference between the open and closed states, their conversion should involve the transformation of the β7/8 sheet and β1 strand. Some experimental data suggests that the open Mad2 state undergoes a conformational change to the closed state via a high-energy intermediate conformation, which might favor Cdc20 binding [26,32]. On the other hand, the theoretical investigations on the Mad2 protein are very limited so far. Therefore, it remains unclear what the dynamical transition mechanism of the two states is and what the exact allosteric network in this conversion is. Because it is difficult to experimentally isolate the intermediate structures during the conformational transition process, it is highly valuable to explore the conformational transition process in more detail by performing computational simulations at the atomic level.

In the present study, we carried out conventional molecular dynamics (CMD) and targeted molecular dynamics (TMD) simulations in order to explore the conformational transition pathway in more detail. The results obtained from the simulations provide valuable insights into this conformational transition process with respect to how one state gradually transforms into another.

## 2. Results

The initial structures of the open and closed Mad2 protein states (denoted as O-Mad2 and C-Mad2, respectively, for convenience) used in the CMD simulations were based on previously reported and deposited X-ray crystal structures (PDB codes 1DUJ and 2VFX (K chain)) [14,23]. The O-Mad2 model consists of three α helices (αA: 18–37, αB: 61–75, and αC: 122–142) and eight β strands (β1: 11–13, β2: 43–49, β3: 53–58, β4: 81–90, β5: 97–105, β6: 150–160, β7: 170–174 and β8: 184–191). The missing residues (Met1–Gly10, Met196–Asp205) in this model were built using the loop search method in the Swiss-Pdb Viewer (http://spdbv.vital-it.ch/). The C-Mad2 model consists of three α helices (αA: 12–37, αB: 61–75, and αC: 122–142) and seven β strands (β2: 43–49, β3: 53–58, β4: 81–90, β5: 97–105, β6: 150–160, β8′: 177–188, and β8″: 190–200). The mutated residue Ala13 in the C-Mad2 model was modeled by starting from the original residue Leu13.

**Figure 1.**
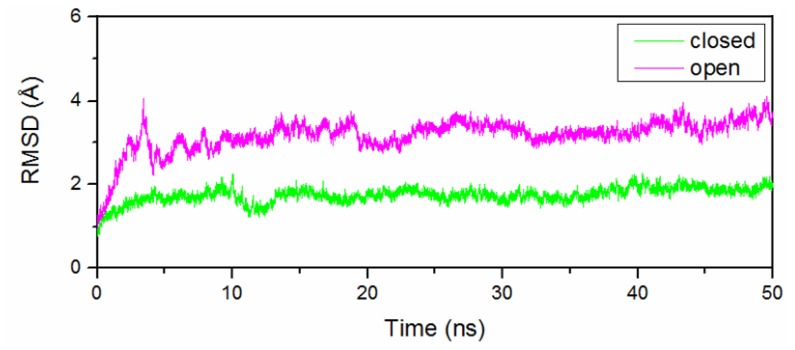
Root mean square deviation (RMSD) values of all backbone atoms of the O-Mad2 and C-Mad2 models with respect to the corresponding starting structure for the simulations of O-Mad2 and C-Mad2 models.

**Figure 2.**
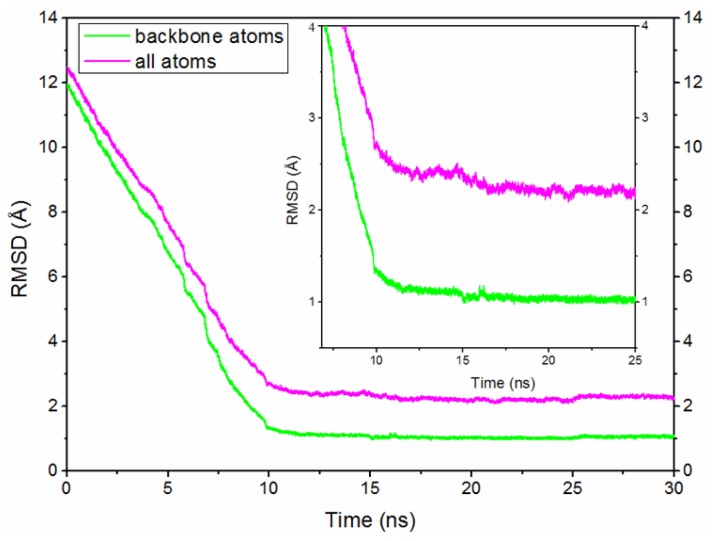
Root mean square deviation (RMSD) values of all backbone atoms and all atoms of the whole protein are shown for the O-Mad2→C-Mad2 transition.

To explore the detailed conformational transition pathway, we first carried out CMD simulations on the O-Mad2 and C-Mad2 states to understand the stability of the two Mad2 structures, followed by TMD simulations associated with the transition from the open state to the closed state and *vice versa* to explore the dynamic transition between the two states of the protein. Depicted in Figure 1 are the plots for the time-dependent root mean square deviations (RMSDs) for the positions of all backbone atoms in the CMD-simulated O-Mad2 and C-Mad2 structures, revealing that the CMD trajectories tended to be stabilized after about 5 ns; the corresponding energies also tended to be stabilized after about 5 ns. Therefore, for each protein state, trajectory analysis was performed to extract the equilibrated conformation between 30 and 50 ns simulation time, recording 10,000 snapshots (one for each 2 ps) for each trajectory. Depicted in Figures 2 and S1 are the plots for the RMSDs of the positions of all backbone atoms in the TMD-simulated protein structures from the corresponding final structures associated with the two TMD trajectories for the O-Mad2↔C-Mad2 conversion. The plots depicted in Figures 2 and S1 reveal that the backbone atoms in the two TMD simulations all reached the target conformations within 12 ns. The detailed structural transition results obtained from the CMD and TMD simulations are discussed below in detail.

### 2.1. Stability of the O-Mad2 and C-Mad2 States

**Figure 3.**
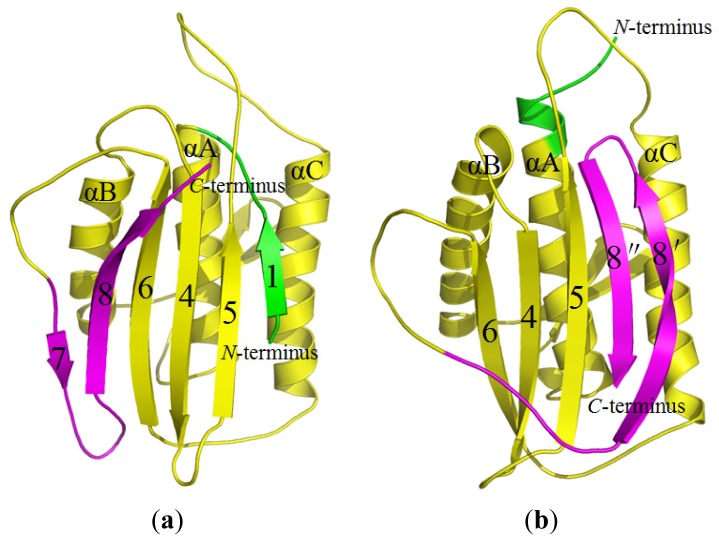
MD-simulated Mad2 structures for the open state (**a**) and closed state (**b**).

Based on the CMD simulations (see Figure 1), we obtained two dynamically stable states (the open and closed states) of Mad2. For each state, the average structure extracted from the CMD trajectory is shown in Figure 3. According to our analysis on the simulated structures, both the O-Mad2 and C-Mad2 structures simulated reflect the characteristics of the X-ray crystal structures very well. For example, these two states consist of three layers: a central layer formed by three α-helices, a large six- or five-stranded β-sheet in the front layer, and a short β-hairpin in the back layer. Specifically, in the O-Mad2 state, the three α-helices in the central layer are αB–αA–αC, a large β7–β8–β6–β4–β5–β1 β-sheet in the front layer, and a short β2–β3 β-hairpin in the back layer arranged orderly from left to right in Figure 3a. In the C-Mad2 state, except for the large β6–β4–β5–β8″–β8′ β-sheet rearranged in the front layer, similar components in the central and back layers can be found in Figure 3b. The most remarkable differences between the O-Mad2 and C-Mad2 states are: (1) the β7/8 sheet is located on the left side of the β-sheet in O-Mad2 and the β8″/8′ sheet located to the right in C-Mad2; (2) the β1 strand in O-Mad2 forms a new helix attached to the *N*-terminus of αA in C-Mad2.

### 2.2. The Conformational Transition from O-Mad2 to C-Mad2

**Figure 4.**
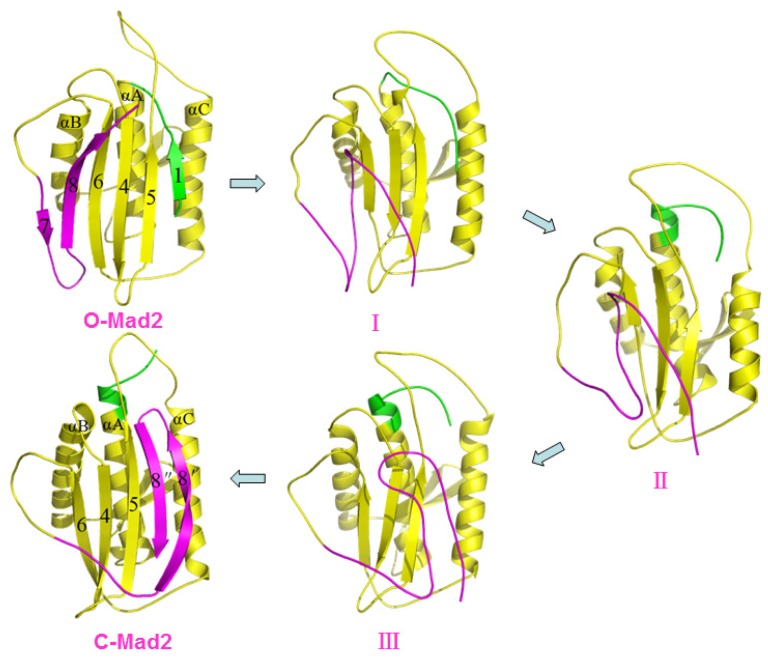
The three-dimensional structures of the conformational transition pathway determined by TMD from O-Mad2 to C-Mad2.

Based on the structural features of the metamorphic protein Mad2, we performed TMD simulations to explore the conformational transition process between O-Mad2 and C-Mad2. Based on the two TMD simulations for the O-Mad2↔C-Mad2 conversion, the forward and reverse transitions almost followed similar structural conversion processes computationally (see Figures 2, 4, S1 and S2). Consequently, we only present the conformational transition from O-Mad2 to C-Mad2 starting from the equilibrated O-Mad2 structure. During the transition process, three transition conformations (I (~3.5 ns), II (~6.5 ns), III (~9.5 ns)) of the Mad2 protein were extracted from the TMD simulation trajectory and were simulated via the CMD method as initial structures. The corresponding time-average structures are shown in Figure 4. In the first step of the transition (from O-Mad2 to I), the β1 strand in the O-Mad2 state extends to a line loop and, simultaneously, the β7/8 sheet unfolds to form conformation I with the destruction of their hydrogen bonds and hydrophobic interactions. In the second step of the transition (from I to II), the unfolded β1 strand in conformation I traverses through the β5-αC loop close to the αA helix and forms a new helix attached to the *N*-terminus of αA helix to produce conformation II, which causes the large fluctuation of the β5-αC loop and favors the β7/8 shift through emptying the right region of the β6/4/5 sheet. Simultaneously, the β6/4/5 sheet in the front layer moves close to the αC helix in the central layer (see Figure 4), which results in the exposure of binding sites around the β6 strand and an increase in the space between the β6 strand and the unfolded β7/8 sheet, ultimately increasing its activity to combine with the Cdc20 protein. Because the structural characteristics of II showed that the new helix, attached to the *N*-terminus of αA helix is completely formed and that the β6/4/5 sheet is closest to the αC helix in the central layer (see Discussion Section), II was regarded as the intermediate during the conversion of O-Mad2 to C-Mad2. In the third step, *i.e.*, the conformational transition from II to III, the unfolded β7/8 sheet in conformation II moves from the left side of β6/4/5 sheet to its front to produce conformation III. In the fourth step of the transition (from III to C-Mad2), the unfolded β7/8 sheet in conformation III moves to the right side of the β6/4/5 sheet to form a new β8″/8′ sheet and produces the C-Mad2 state through formation of new hydrogen bonds and hydrophobic interactions (see Figure 4). In summary, the transition process from O-Mad2 to C-Mad2 involves the initial unfolding of the β7/8 sheet followed by the transfer of the unfolded β7/8 sheet. Moreover, the β1 strand traversing through the β5-αC loop is a prerequisite for β7/8 translocation. The intermediate II is characterized by the exposure of the β6 strand and the increase in space of binding sites around β6, which is possibly a favorable conformation to combine with the Cdc20 protein to mediate APC/C inhibition.

### 2.3. Correlation Analysis along the Transition Pathway from O-Mad2 to C-Mad2

**Figure 5.**
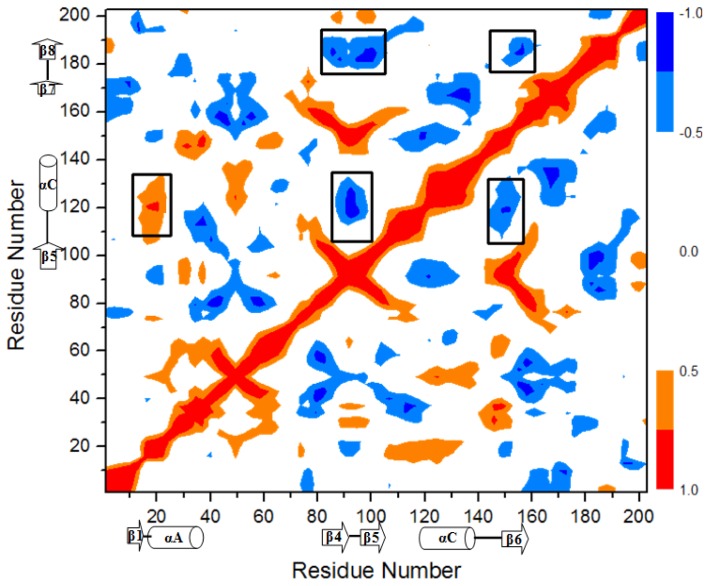
Dynamical cross-correlation map of the transition from O-Mad2 to C-Mad2 with specific sub-region squares in black.

To explore the allosteric communication of position changes for the β1 strand and the β7/8 sheet along the transition pathway from the O-Mad2 state to the C-Mad2 state, motion correlations of Cα atoms were analyzed and the corresponding cross-correlation map constructed from the trajectory is given in Figure 5 for the conversion of O-Mad2→C-Mad2. The results showed that the motion correlations between the residues ranged from high anticorrelation (blue) to high correlations (red). The results in Figure 5 demonstrate that the large correlated motions of the β1 strand *vs*. the β5-αC loop occur remarkably with the large correlated motions of the β5-αC loop *vs*. the β6/4/5 sheet, which predicts that the allosteric communication between the β1 strand and the β6/4/5 sheet occurs via the β5-αC loop region. Furthermore, the motions of the β6/4/5 sheet significantly correlate with the motions of the β7/8 sheet, which predicts the allosteric communication between the β6/4/5 and the β7/8 sheets.

## 3. Discussion

### 3.1. Conformational Characteristics along the Transition Pathway from O-Mad2 to C-Mad2

**Figure 6.**
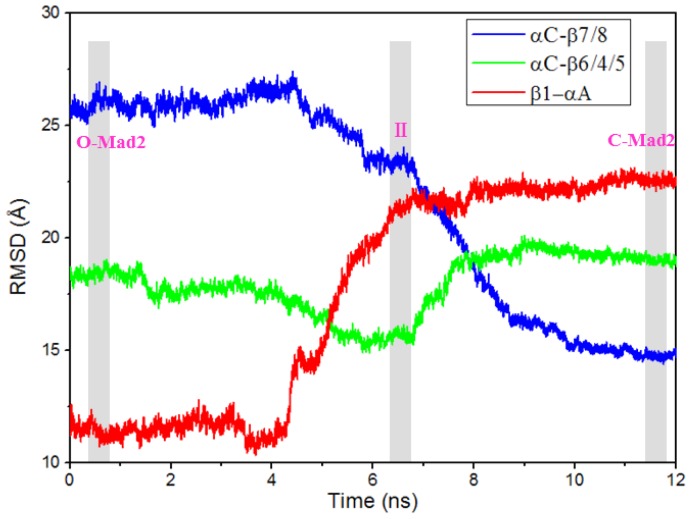
Mass center distances between the β6/4/5 sheet and αC helix (green), between the β7/8 sheet and αC helix (blue), and between the β1 strand and αA helix (red) along the transition pathway from O-Mad2 to C-Mad2. The roseate letters included in the grey areas denote the different conformations.

**Figure 7.**
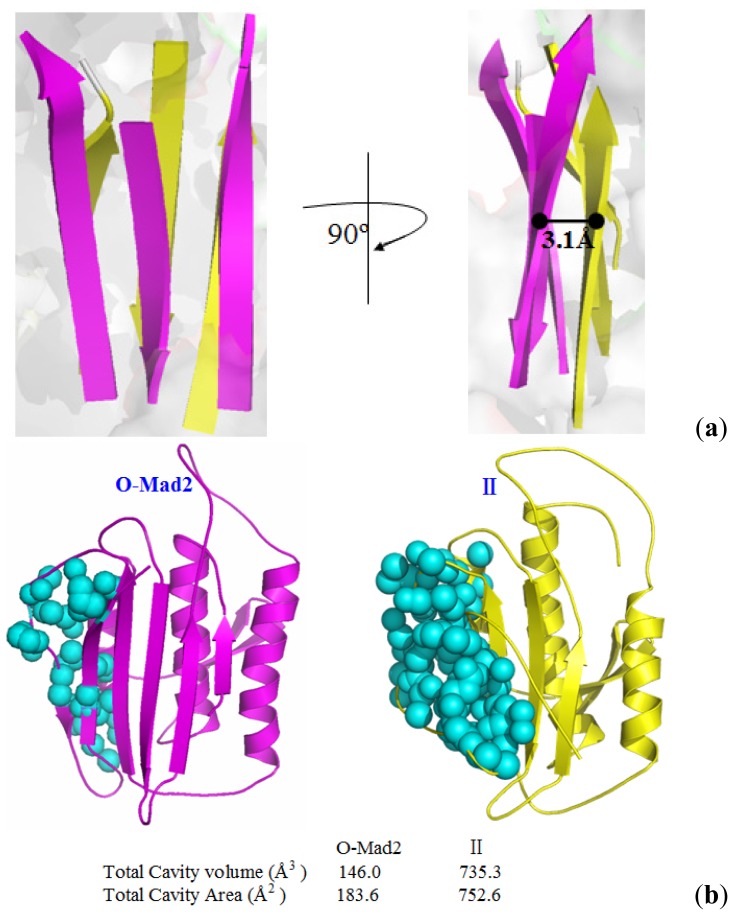
(**a**) Moving distance of the β6/4/5 sheet between O-Mad2 and II; and (**b**) representation of the pockets detected by the CASTp program (cyan spheres: Cdc20 binding site pocket).

To explore the details of the structural changes along the transition pathway from O-Mad2 to C-Mad2, we used the αA and αC helices as reference points to calculate the variations in mass center distances between the β1 strand and the αA helix, between the β7/8 sheet and the αC helix, and between the β6/4/5 sheet and the αC helix. The corresponding results are shown in Figure 6. It can be seen from the conversion of O-Mad2 to C-Mad2 that the distance between the β1 strand and the αA helix increases from 11.4 to 22.5 Å, which corresponds to the β1 strand traversing through the β5-αC loop and the emptying the right region of the β6/4/5 sheet, consequently favoring the β7/8 translocation. Moreover, the distance between the β7/8 sheet and the αC helix decreases from 26.2 Å in the O-Mad2 state to 14.8 Å in the C-Mad2 state, which represents the movement of the β7/8 sheet from the left side of the β6/4/5 sheet to its right side. Interestingly, this distance variation is slower than that between the β1 strand and the αA helix (see Figure 6), which suggests that the β1 strand traversing through the β5-αC loop occurs prior to the β7/8 translocation, as discussed in Section 2.2. In particular, in the conformational transition from O-Mad2 to II, the distance between the β6/4/5 sheet and the αC helix decreases from 18.1 Å in the O-Mad2 state to 15.0 Å in the intermediate II, which corresponds to the movement of the β6/4/5 sheet in the front layer closest to the αC helix in the central layer to form the intermediate. As expected, the moving distance of the β6/4/5 sheet with 3.1 Å predicts that the Cdc20 binding sites, *i.e.*, residues Leu61, Tyr64, Asn67, Trp75, Asp152–Tyr156, Asp158, Asp160–Leu161, Val163, Lys166–Glu169, Pro172, Phe174 around the β6 strand [24], in the intermediate could be considerably exposed compared with the O-Mad2 state (see Figure 7a). To further address the exposed space of Cdc20 binding sites in the intermediate, the CASTp program was used to calculate the size and shape of its pocket compared with that in the O-Mad2 state. Such analysis was shown to be able to provide a comprehensive and detailed quantitative characterization of interior voids and surface pockets of proteins [33–35]. Figure 7b shows the binding site pockets in the O-Mad2 state and the intermediate, which are automatically identified by the CASTp analysis. The two topological equivalent cavities for the binding site pockets in the O-Mad2 state and the intermediate have different areas and volumes of 183.6 Å^2^/146.0 Å^3^ and 752.6 Å^2^/735.3 Å^3^, respectively. This result reveals that the size of the binding site pocket in the intermediate is much larger than in O-Mad2, which suggests that the intermediate is a favorable conformation for Cdc20 protein binding in the mitotic process. However, in the conformational transition from intermediate II to C-Mad2, the distance between the β6/4/5 sheet and the αC helix increases from 15.0 to 19.8 Å, which increases the locating space for the newly formed β8″/8′ sheet and favors the β7/8 translocation to form the β8″/8′ sheet.

### 3.2. Hydrogen Bond and Hydrophobic Structure Analyses along the Transition Pathway from O-Mad2 to C-Mad2

**Figure 8.**
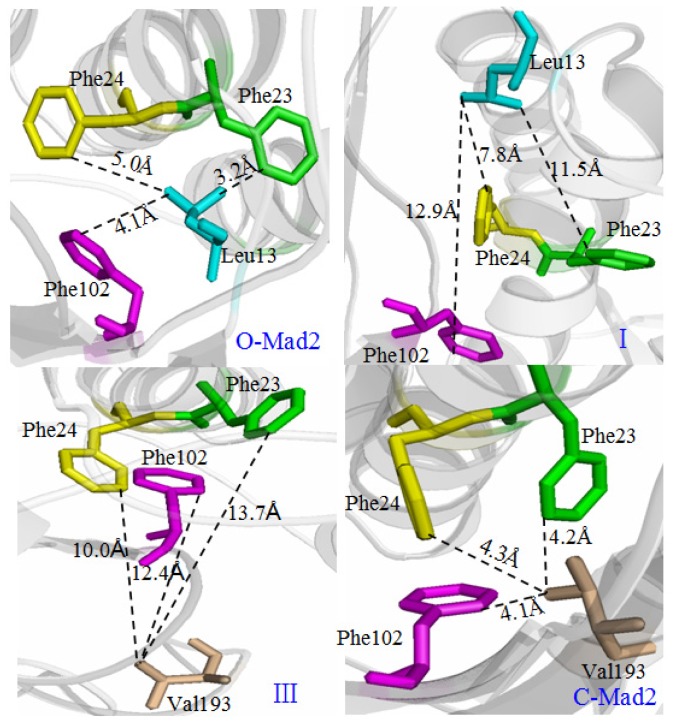
Representation of the hydrophobic interaction in the conformational transition pathway from O-Mad2 to C-Mad2.

To gain information of hydrogen bonds and the hydrophobic core of Mad2, analyses of hydrogen bonds and hydrophobic contacts in the conversion process of O-Mad2 to C-Mad2 were performed. The occurrence of all possible hydrogen bonds for the β1 strand and the β7/8 sheet in the conversion process are shown in Table 1 by calculating the percentages of times on the corresponding CMD simulations. Based on the experimental observations, the hydrophobic core involves the residues Leu13 in β1, Phe23 and Phe24 in αA, Phe102 in β5, and Val193 in β8″ [25]. Figure 8 shows the hydrophobic contacts at the hydrophobic core. A hydrophobic contact is defined as a distance between carbon atoms shorter than 4.5 Å. For the conformational transition from O-Mad2 to II, based on the unfolding of the β1 strand and the β7/8 sheet in the step of O-Mad2 to I (as discussed in Section 2.2), some hydrogen bonds that initially exist in the O-Mad2 state disappear gradually with the conformational change to I. For example, the hydrogen bond between the N–H group of Asp101 in β5 and the O atom of Gln9 in β1 is maintained with the occupancy of 98% of simulation times in the O-Mad2 state, while the same hydrogen bond is maintained with the occupancy of ~0% of simulation times in conformation I. Moreover, visual analysis shows that the hydrophobic contact distances of 3.2, 5.0 and 4.1 Å between Leu13 and Phe23, between Leu13 and Phe24, and between Leu13 and Phe102 in the O-Mad2 state increase to 11.5, 7.8 and 12.9 Å, respectively, which predicts that hydrophobic interaction destruction occurs because Leu13 moves away during β1 unfolding (see Figure 8). In the formation of the new helix at the *N*-terminus of the αA helix in transition from I to II, as discussed, some hydrogen bonds are formed with the conformational change to II. For example, the hydrogen bond between the N–H group of Ser16 and the O atom of Thr12 is maintained with the occupancy of 98.8% of simulation times in intermediate II, while this hydrogen bond is maintained with the occupancy of ~0% of simulation times in conformation I.

For the conformational transition from II to C-Mad2 in the formation of the new β8″/8′ sheet in the transition of III to the C-Mad2 state, as discussed in Section 2.2, some new hydrogen bonds are formed with the conformational change to the C-Mad2 state. For example, the hydrogen bond of NH(Asp103)–O(Asn194) in the β8″/8′ sheet with the occupancy of 94.2% of simulation times appears in the C-Mad2 state. Moreover, visual analysis shows that the hydrophobic contact distances of 13.7, 10.0, and 12.4 Å between Val193 and Phe23, between Val193 and Phe24, and between Val193 and Phe102 decreases to 4.2, 4.3, and 4.1 Å, respectively, in the C-Mad2 state, which predicts that a new hydrophobic interaction is formed with Val193 in the newly formed β8″ moving into this hydrophobic core (see Figure 8).

### 3.3. Allosteric Network Analysis in the Conversion of O-Mad2 to C-Mad2

**Figure 9.**
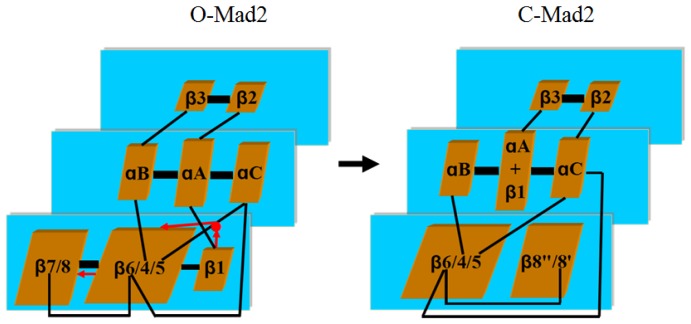
Color coded optimal correlated segment network of O-Mad2 (**left**) and C-Mad2 (**right**). Widths of the connecting black-lines are proportional to the intersegment edges. The red-lines with arrowheads and the red dot represent the correlation motion connections and the mass center of the β5-αC loop, respectively.

To fully understand the allosteric communication in the conversion of the O-Mad2 state to the C-Mad2 state, we explored the relationship between the motion correlations and their conformational changes. It can be seen from the motion correlation analysis in Figure 5 that the β1 strand correlates to the β5-αC loop with large cross-correlate coefficients. Simultaneously, the β5-αC loop correlates to the β6/4/5 sheet; then the β6/4/5 sheet further correlates to the β7/8 sheet. Such communication transition represents the allosteric communication network from the β1 strand to the β7/8 sheet through the β5-αC loop and β6/4/5 sheet. A coarse-grained picture of correlated segments of O-Mad2 and C-Mad2 is shown in Figure 9 with correlation connections. The width of the black-lines connecting correlated segments is proportional to the sum of the edges connecting them. The red-lines with arrowheads represent the correlation connections. It can be seen that the segment of the β1 strand in the O-Mad2 state merges into the new segment of β1 + αA in the C-Mad2 state, which induces the fluctuation of the β5-αC loop due to the fact that the β1 strand traverses to the *N*-terminus of the αA helix through the β5-αC loop. Such allosteric motion produces the correlation connection between the β1 strand and the β5-αC loop. Because the β5 strand and the αC helix are connected to the β6 strand and the β4 strand, respectively, the movement of the β6/4/5 sheet to the αC helix relates definitely to the β5-αC loop, as expected for the correlations of the β5-αC loop to the β6/4/5 sheet. Furthermore, the motion of the β6/4/5 sheet facilitates the translocation of the β7/8 sheet due to the connection of the β6/4/5 sheet to the β7/8 sheet, subsequently causing allosteric communication to the β7/8 sheet.

## 4. Models and Methods

### 4.1. Conventional Molecular Dynamics Simulation

All CMD simulations for these built models were carried out using the AMBER 9 package [36] and ff03 all atom force field parameters [37–39]. Five Na^+^ ions were used to neutralize each of the O-Mad2 and C-Mad2 models, and an ionic strength of 50 mM was generated by adding 9 Na^+^ and 9 Cl^−^ ions for these two models [14]. Similar counterion processes were applied to the other models. Each of the systems was explicitly solvated by using the TIP3P water potential inside an orthorhombic box of water molecules with a minimum solute-wall distance of 10 Å. The protocol for all CMD simulations is described as follows: (1) the systems were energetically minimized to remove unfavorable contacts. Four cycles of minimizations were performed with 5000 steps of each minimization and harmonic restraints on the Mad2 protein from 100, 75, 50 to 25 kcal/(mol·Å^2^), which means that the restraints were relaxed stepwise by 25 kcal/(mol·Å^2^) per cycle. The fifth cycle consists of 10,000 steps of unrestrained minimization before the heating process. The cutoff distance used for the non-bonded interactions was 10 Å. The SHAKE algorithm [40] was used to restrain the bonds containing hydrogen atoms; (2) Each energy-minimized structure was heated over 120 ps from 0 to 300 K (with a temperature coupling of 0.2 ps), while the positions of Mad2 protein were restrained with a small value of 25 kcal/(mol·Å^2^). The constant volume was maintained during the processes; (3) The unrestrained equilibration of 200 ps with constant pressure and temperature conditions was carried out for each system. The temperature and pressure were allowed to fluctuate around 300 K and 1 bar, respectively, with the corresponding coupling of 0.2 ps. For each simulation, an integration step of 2 fs was used; and (4) Finally, conventional molecular dynamics (CMD) runs of 50 ns for the C-Mad2 and the O-Mad2 models were carried out, respectively, by following the same protocol.

### 4.2. Targeted Molecular Dynamics Simulation

Targeted MD simulation [41,42] is a method to observe large-scale conformational transitions between two known end-point conformations of a molecule. A restraint energy term was added to the energy function proportional to the square of the difference, which may be characterized as the mass-weighted root-mean-square deviation (RMSD) of the current structure to the target structure in terms of atomic positions. The functional form of the restraint energy can be written as:

(1) $$E_{\text{TMD}}=\frac{1}{2}kN{\left[RMSD(t)-RMSD_{0}(t)\right]}^{2}$$

where, *k* is the harmonic force constant per atom, *N* is the number of the restrained atoms, *RMSD* (*t*) is the root-mean-square deviation of the simulated structure at time *t* relative to the target structure, and *RMSD*_0_ (*t*) is the prescribed target *RMSD* value at time *t* that decreases to zero linearly with time to drive the system from an initial structure to the target structure. To obtain the appropriate small force constant *k*, four independent, short (4 ns) TMD simulations were performed using different force constants of 0.2, 0.5, 0.8 and 1.0 kcal/(mol·Å^2^), respectively. As shown in Figure S3 for the conversion of O-Mad2 to C-Mad2, three out of four simulations reached a *RMSD* value of <2.0 Å extracting from the backbone atoms of the Mad2 protein. Therefore, *k* = 0.5 kcal/(mol·Å^2^) was chosen as the lowest harmonic force constant to apply onto all the backbone atoms of the Mad2 protein to bias the trajectories toward the target structure. All the other atoms in the Mad2 protein were assumed to move freely and to rearrange at their convenience to let the protein reach its equilibrium under this constraint. The weighted RMSDs along the TMD trajectories from four, 4 ns independent simulations showed that each process could follow the same conformational transition pathway, which indicated that the external forces and initial velocities did not affect the transition pathway. Therefore, the final TMD simulations were performed with the 10 ns transition TMD, followed by 20 ns of equilibration with an integration step of 2 ps. To test the mutual conversion property between the O-Mad2 and C-Mad2 states, the TMD simulations were performed in an O-Mad2↔C-Mad2 bidirectional fashion.

### 4.3. Correlation of Atomic Motions

The dynamic feature of a protein and the extent of correlation of motions in the different regions of a protein were assessed *via* the calculation of cross-correlation coefficients, C(*i*,*j*), as follows:

(2) $$C(i,j)=(\Delta r_{i}\times \Delta r_{j}/{\left[(\Delta {r_{i}}^{2})(\Delta {r_{j}}^{2})\right]}^{1/2}$$

In the equation, Δ*r_i_* and Δ*r_j_* are the displacement vectors for Cα atoms of residues *i* and *j*, respectively, and the angle brackets denote the ensemble averages [36,43]. In the present study, the correlation coefficients were averaged over the regions of protein, and the resulting cross-correlation coefficients are presented in the form of a two-dimensional graph. These structure analyses in the present work were calculated by using the PTRAJ module of the AMBER 9 program.

### 4.4. Surface Analyses

Shape descriptors representing protein structure, such as depth, surface curvature, extreme elevation, surface area, and volume, have been used extensively to identify, study, and compare protein-ligand interactions, protein-protein interactions, and the respective binding sites. We used the Computed Atlas of Surface Topography of proteins (CASTp) program (http://sts.bioengr.uic.edu/castp/) [44] to determine the binding pocket. The CASTp program uses weighted Delaunay triangulation and the alpha complex for shape measurements. It provides identification and measurements of surface accessible pockets as well as interior inaccessible cavities for proteins and other molecules. It analytically measures the area (in Å^2^) and volume (in Å^3^) of each pocket and cavity, both in solvent accessible surface (SA, Richards’ surface) and molecular surface (MS, Connolly’s surface) [44]. In our work, the probe radius was set to the default value (1.4 Å). The detected pockets from using these algorithms are ranked with their volumes and areas. For ligand molecules, internal protein cavities appear to be a favored binding site, and the cavity volume may play an important role in the strength of the guest molecule–host cavity interaction. In CASTp analysis, we chose the Cdc20-binding pockets for our analysis.

## 5. Conclusions

Conventional molecular dynamics simulations and targeted molecular dynamics simulations were performed to address the stable structure characteristics for the open and closed Mad2 states and the dynamic transition mechanism of the two states, respectively. The results of this study show that the predominant structural differences between the stable open and closed Mad2 states were the transformations of the β7/8 sheet and the β1 strand with the rearrangement of β7–β8–β6–β4–β5–β1 to β6–β4–β5–β8″–β8′. The results for the transition mechanism investigation demonstrated that the structural transition from the open Mad2 state to the closed one could occur via an intermediate. The structural change characteristics along the transition pathway indicate that the β1 strand and the β7/8 sheet unfold first, then move, and finally form an additional helix and a new β8″/8′ sheet via the intermediate. Particularly, the β1 strand traversing through the β5-αC loop is a prerequisite for the β7/8 translocation. The conformation analysis for the intermediate indicates that the movement of the β6/4/5 sheet close to the αC helix causes the exposure of the β6 strand and an increase in size of the active pocket, and favors the binding of the Mad2 protein to Cdc20. This observation reveals the critical function of interconversion of the two states in APC/C inhibition. Correlation analysis reveals that the allosteric network occurs from the β1 strand to the β7/8 sheet via the β5-αC loop and the β6/4/5 sheet in the conversion of the open Mad2 state to the closed Mad2 state. The present investigation provides useful insights into understanding the dynamics of the transition mechanism of the active closed Mad2 state to the open Mad2 state.

## Supplementary Information



## Figures and Tables

**Table 1.** The occupancies (%) of hydrogen bonds for the O-Mad2 state, I, II, III, and C-Mad2 state.

Hydrogen bond	O-Mad2	I	II	Hydrogen bond	III	C-Mad2
(Ile11)N–H···O(Gln101)	98.2	0.0	–	(Arg99)N–H···O(Ala198)	0.0	99.6
(Gln101)N–H···O(Gln9)	98.0	0.0	–	(Arg99)O···H–N(Ala198)	0.0	95.6
(Asp103)N–H···O(Ile11)	83.2	0.0	–	(Gln101)N–H···O(Met196)	0.0	100.0
(Phe151)N–H···O(Ser185)	98.0	0.0	–	(Gln101)O···H–N(Met196)	0.0	98.8
(Leu153)N–H···O(Thr187)	87.6	0.0	–	(Asp103)N–H···O(Asn194)	0.0	94.2
(Ile155)N–H···O(Thr189)	98.8	0.0	–	(Asp103)O···H–N(Asn194)	0.0	99.6
(Thr157)N–H···O(His191)	89.8	0.0	–	(Glu105)N–H···O(Lys192)	0.0	98.4
(Thr188)N–H···O(Pro172)	56.6	0.0	–	(Glu105)O···H–N(Lys192)	0.0	100.0
(Thr189)N–H···O(Leu153)	99.4	0.0	–	(Glu179)N–H···O(Tyr199)	0.0	99.4
(His191)N–H···O(Ile155)	99.4	0.0	–	(Glu179)O···H–N(Tyr199)	0.0	98.6
(Thr187)N–H···O(Phe151)	90.6	0.0	–	(Val181)N–H···O(Val197)	0.0	98.6
(Phe186)N–H···O(Phe174)	88.0	0.0	–	(Val181)O···H–N(Val197)	0.0	92.4
(Phe186)O···H–N(Phe174)	97.8	0.0	–	(Leu183)N–H···O(Ser195)	0.0	99.4
(Ser16)N–H···O(Thr12)	–	0.0	98.8	(Phe186)N–H···O(Val193)	0.0	99.4
(Ala17)N–H···O(Leu13)	–	0.0	85.7	(Phe186)O···H–N(Val193)	0.0	100.0
(Glu18)N–H···O(Arg14)	–	0.0	57.9	(Thr188)N–H···O(His191)	0.0	94.2
(Ile19)N–H···O(Gly15)	–	0.0	88.4	(Lys200)N–H···O(Leu97)	0.0	94.2
(Val20)N–H···O(Ser16)	–	0.0	99.2			
(Ala21)N–H···O(Ala17)	–	0.0	54.5			
