# Supplementary material for: Molecular Dynamics Simulation on the Conformational Transition of the Mad2 Protein from the Open to the Closed State
Source: Int J Mol Sci. 2014 Mar 31;15(4):5553–69. doi: 10.3390/ijms15045553 (PMC4013581; doi:10.3390/ijms15045553)
Supplement: Supplementary file 1 [file ijms-15-05553-s001.pdf]

Supplementary Information

Figure S1. RMSD values of all backbone atoms and all atoms of the whole protein are shown for the C-Mad2→O-Mad2 transition.

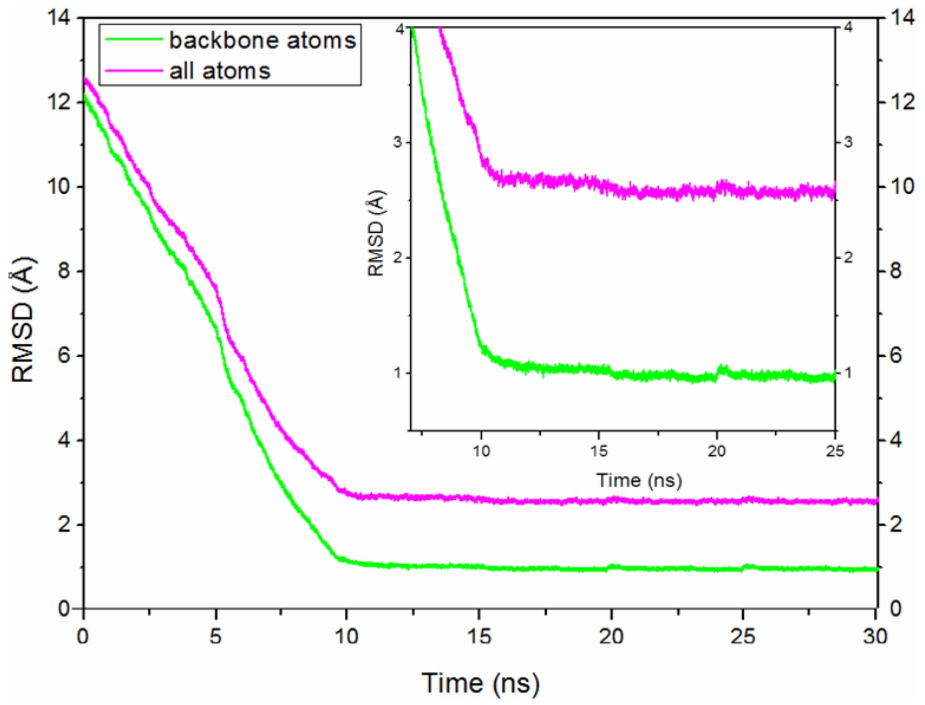

Figure S2. The three-dimensional structures of the conformational transition pathway determined by TMD from C-Mad2 to O-Mad2.

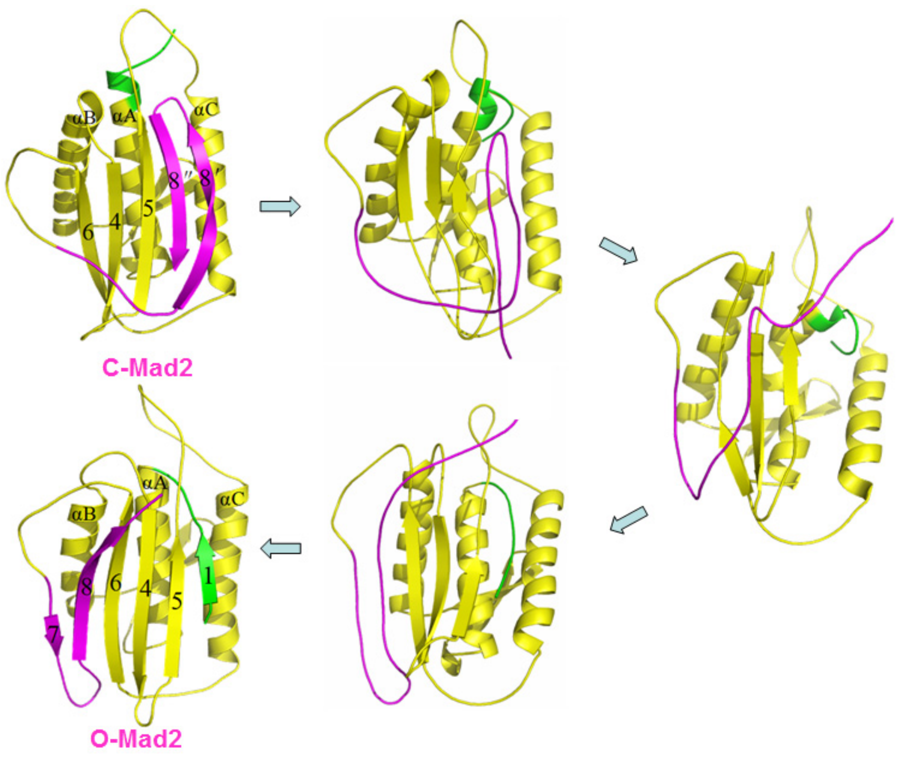

Figure S3. RMSD value of backbone atoms for the conversion of O-Mad2 to C-Mad2 using varying force constants *k*, in kcal/(mol·Å^2), shown in the inset.

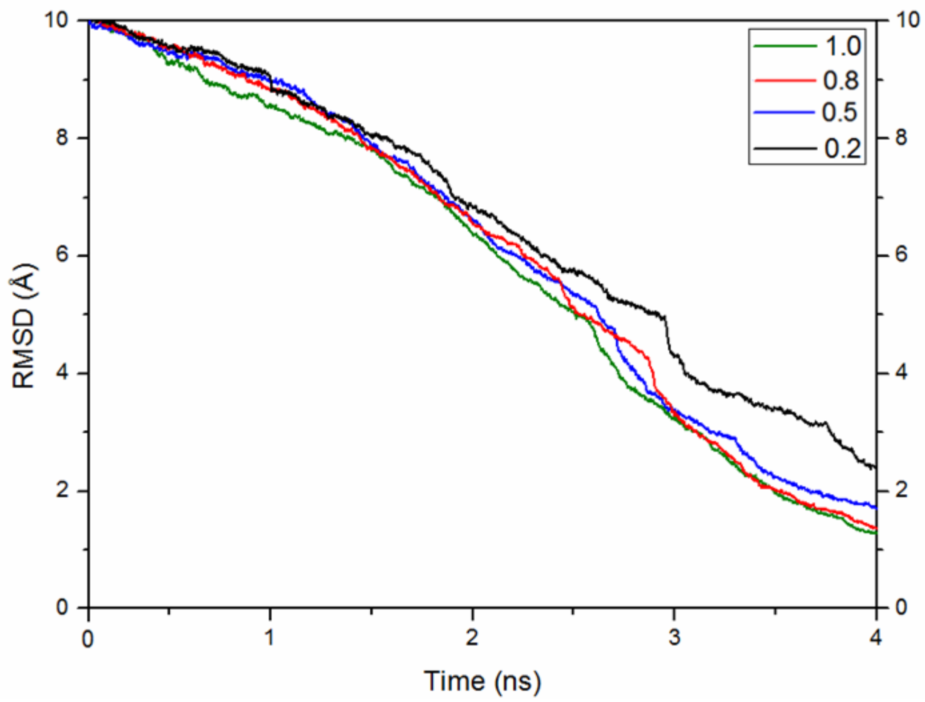

© 2014 by the authors; licensee MDPI, Basel, Switzerland. This article is an open access article distributed under the terms and conditions of the Creative Commons Attribution license (http://creativecommons.org/licenses/by/3.0/).
